# Chlorothalonil exposure impacts larval development and adult reproductive performance in *Drosophila melanogaster*

**DOI:** 10.1098/rsos.250136

**Published:** 2025-06-04

**Authors:** Darshika M. Dissawa, Ines Boyer, Fleur Ponton

**Affiliations:** ^1^ School of Natural Sciences, Macquarie University, Sydney, New South Wales, Australia; ^2^ PURPAN Engineering School, Toulouse, France

**Keywords:** pesticide, insect, agrochemical, non-target organism, chronic exposure, fitness

## Abstract

Chlorothalonil, a widely used fungicide in agriculture, has raised concerns regarding its impact on non-target species. This study examined the effects of chronic chlorothalonil exposure on larval development and reproductive performance of the vinegar fly, *Drosophila melanogaster*. Drosophila eggs were reared on a diet supplemented with sublethal concentrations of chlorothalonil (5–120 mg kg^−1^). Larval pupation was measured as an indicator of survival and growth, while fecundity, ovariole count and body weight served as proxies for female reproductive performance. A ferrozine assay was used to examine mitochondrial mitoferrin activity in males as a reproductive marker related to iron metabolism. Chlorothalonil exposure decreases larval survival, extends developmental duration and reduces fecundity. Even at the lowest tested concentration, chlorothalonil exposure resulted in reduced body weight, ovariole count and egg production compared with non-exposed individuals. Male flies also demonstrated reduced iron levels. These findings underscore that chronic, sublethal chlorothalonil exposure not only induces larval mortality but also adversely affects fecundity in adult insects. Assessing the toxicological effects of agrochemicals on non-target organisms is critical to understanding the broader environmental impacts of these substances. Such insights are vital for developing conservation strategies that safeguard ecosystems and support biodiversity while promoting sustainable agricultural practices.

## 1. Introduction

The global decline of insect populations has become a critical global concern [1,2]. Studies across various ecosystems have documented significant reductions in insect biomass, with certain regions in Europe and North America experiencing declines exceeding 75% in recent decades [3,4]. This trend is not restricted to specific geographic regions or taxa but spans from pollinators to decomposers and other functional groups that play essential roles in ecosystem services [5–7]. The rapid pace and scale of these declines raise concerns about cascading ecological effects and the risk of destabilizing both natural and agricultural systems [8,9].

A complex interplay of factors drives these insect declines, with primary contributors including habitat loss, climate change, invasive species, pollution and notably, pesticide use [8,10]. Pesticides have gained considerable attention as they are extensively used in agriculture and can exert both lethal and sublethal effects on non-target insect species [11,12]. Modern agricultural practices often involve high rates of pesticide application across landscapes, exposing insects to various chemical classes, including insecticides, herbicides and fungicides [13–15]. Insecticides are specifically designed to target pest insects. However, other classes, such as fungicides and herbicides, although primarily aimed at fungi and plants, can also indirectly impact insect populations by contaminating habitats and food sources or through chemical interactions with other agrochemicals [16–20]. The broad-reaching effects of pesticides highlight the importance of understanding their role in the broader decline of entomofauna [10].

Fungicides, formulated primarily to inhibit fungal growth, represent a significant yet overlooked class of agrochemicals regarding their effects on insect populations [16,17]. While fungicides are often perceived as less harmful to insects than insecticides, recent studies suggest they may exert considerable non-target effects on insect physiology, immunity and behaviour, indirectly disrupting ecological interactions, such as plant–insect relationships, with potential consequences for pollination and biodiversity [21–25]. For example, fungicides can disrupt the gut microbiota in bees, impairing immune function and increasing disease susceptibility [26]. In agricultural systems, fungicides are often applied as prophylactic treatments alongside insecticides, leading to combined or synergistic exposure risks [26–28].

Chlorothalonil, a widely used broad-spectrum fungicide, is especially concerning due to its extensive application on crops like cereals, vegetables and fruits to control fungal diseases [29–31]. Chlorothalonil is one of the most extensively applied fungicides globally [32–34]. Chlorothalonil’s persistence in the environment and its mobility have led to its frequent detection in soil and water bodies near agricultural areas, raising concerns about its potential effects on non-target species [35–38]. Recent research suggests that chlorothalonil may pose greater ecological risks than previously understood, with evidence indicating it can adversely affect both vertebrate and invertebrate species, thereby contributing to biodiversity declines [39–42].

Chlorothalonil’s effects on vertebrate species, particularly amphibians, are well documented [43–46]. Research has shown that even low environmental concentrations of chlorothalonil can result in high mortality rates in amphibians, affecting both larval and adult stages. This fungicide disrupts amphibian immune and endocrine systems, increasing susceptibility to pathogens, including *Batrachochytrium dendrobatidis* [39,43–45,47,48]. Furthermore, chlorothalonil exposure has been associated with developmental abnormalities in amphibians, such as stunted growth and limb deformities, which can negatively impact survival and reproduction [39,43–45,47–49]. Amphibians face multiple stressors, including habitat loss and climate change, which could exacerbate fungicide-induced declines [45,50].

In fish, chlorothalonil exposure leads to oxidative stress, impaired gill function and increased mortality, highlighting its toxicity in aquatic systems near agricultural regions [51–53]. In birds, chlorothalonil exposure may occur indirectly through contaminated prey or plant material. Research suggests that chlorothalonil can bioaccumulate, adversely affecting avian reproduction, behaviour and health [54]. This fungicide’s persistence within food webs and potential for bioaccumulation underscore the necessity of comprehensive risk assessments considering its broader ecological impacts [55].

The toxicity of chlorothalonil also extends to invertebrates, with adverse effects on pollinators, soil-dwelling organisms and aquatic insects vital to ecosystem functionality [22,56,57]. For example, chlorothalonil exposure in bees has been shown to impair immune responses, increasing susceptibility to *Nosema* infections and potentially contributing to colony collapse [58]. Additionally, chlorothalonil disrupts the gut microbiota in bees, compromising nutrient absorption and immune regulation, which decreases survival and health [59–61]. These findings underscore chlorothalonil’s role in pollinator decline, with broader implications for both natural ecosystems and agricultural productivity [62]. In soil-dwelling invertebrates, such as springtails (*Collembola* spp.), chlorothalonil exposure reduces survival rates, potentially disrupting nutrient cycling and soil health [63].

Traditionally regarded as ‘relatively non-toxic’ to a range of animals, including insects, based on LD50 values (e.g. 3700 mg kg^−1^ in mice, greater than 63 mg kg^−1^ in honeybees), chlorothalonil may still present risks to non-target insect species under conditions of frequent exposure, such as those commonly encountered in orchards and other agricultural environments [64,65]. The timing of chlorothalonil application often coincides with flowering periods, increasing exposure for foraging insects. Contaminated pollen and nectar collected by worker bees, for example, are brought back to hives, thereby exposing hive members to the fungicide. Chlorothalonil is also found in fruits and vegetables at concentrations up to 460 mg kg^−1^. Furthermore, chlorothalonil spraying is not strictly controlled, and spillover to surrounding plants is very likely. Chlorothalonil contamination in ripened fruits may lead to consistent exposure during larval development for insect larvae that develop within fruits and vegetables.

Despite substantial research on chlorothalonil’s impacts on vertebrates, relatively few studies have investigated its effects on insects, particularly terrestrial species essential for ecosystem services [64–69]. In this study, we examined the effects of chronic exposure to chlorothalonil on the survival and reproduction of *Drosophila melanogaster* using a commercial formulation of chlorothalonil.

*Drosophila melanogaster* is widely recognized as a valuable model organism in toxicological research due to its genetic tractability, short life cycle and well-documented sensitivity to a broad range of environmental contaminants [70]. Sublethal exposure to various agrochemicals, including herbicides [71], pesticides [72] and fungicides [73], has been shown to delay development and reduce fecundity in *Drosophila*, effects that can ultimately suppress population growth and alter population dynamics in contaminated environments. In agricultural ecosystems, *Drosophila* species play a significant role in nutrient cycling and also serve as key prey for birds, amphibians, reptiles and predatory arthropods, thereby contributing to overall ecosystem stability. Disruptions of *Drosophila* populations may cascade through trophic interactions, adversely affecting predator and parasitoid communities, as well as associated microbial assemblages [74,75]. A decline in populations of flies such as *Drosophila* could also impair key ecosystem services such as decomposition and pollination, thereby reducing agroecosystem resilience and productivity [76]. Given the ecological significance of *Drosophila*, it is important to understand how pesticide exposure influences their development and overall fitness. A deeper understanding of these effects is critical for developing sustainable pest management strategies that minimize non-target impacts and support ecological balance in agricultural landscapes.

To assess the impact of chlorothalonil, chlorothalonil exposure concentrations (0–350 mg kg^−1^) were selected based on residue levels reported in fruits and vegetables (0.1–460 mg kg^−1^). Larvae were reared on chlorothalonil-enriched substrates, and key developmental and physiological parameters were evaluated to determine the chronic effects of exposure. Specifically, we measured larval survival, pupation rates, adult body weight, female reproductive metrics (ovariole number and egg production), male mitochondrial iron metabolism (via ferrozine assays) and larval food intake. The selection of classic life-history parameters was based on their relevance as indicators of overall fitness and population-level consequences of chlorothalonil exposure. These parameters are reliable markers of long-term reproductive success and population viability, providing critical insights into the potential ecological impacts of chronic fungicide exposure in both natural and agricultural ecosystems. This study provides insights into the effects of fungicides on insect population declines and highlights the need for further research into the mechanistic pathways of chlorothalonil toxicity in insects.

## 2. Material and methods

### 2.1. Experimental organism

*Drosophila melanogaster* (Canton-S strain, obtained from the Bloomington Drosophila Stock Center) was maintained in cages to allow overlapping generations. The culture conditions were standardized at 25°C with 65% relative humidity and a 12 h light/12 h dark photoperiod. Flies were reared on a standard sugar–cornmeal yeast medium composed of 100 g l^−1^ brewer’s yeast, 5 g l^−1^ cornmeal, 50 g l^−1^ sugar and 10 g l^−1^ agar, with 3 ml propionic acid and 30 ml nipagin (methyl 4-hydroxybenzoate solution, 100 g l^−1^ in 95% ethanol, Clariant UK Ltd, Pontypridd, UK) added to prevent fungal growth.

### 2.2. Chronic exposure to chlorothalonil of *Drosophila* larvae

The effect of chlorothalonil exposure on *Drosophila* larvae was assessed using a commercial chlorothalonil formulation (suspension concentrate) containing 720 g l^−1^ of chlorothalonil (Surefire Chlortan 720). Chlorothalonil residue levels, as previously documented across various fruits and vegetables post-application, range from 0.1 to 460 mg kg^−1^ (see electronic supplementary material, table S1). Chronic exposure treatments involved a gradient of chlorothalonil concentrations: 0, 50, 100, 150, 200, 250, 300 and 350 mg chlorothalonil per kg of substrate, with five replicates per concentration.

Chlorothalonil was dissolved in distilled water and incorporated into the sugar–cornmeal yeast medium. The volume of chlorothalonil solution added to the diet was standardized to ensure consistency across treatments. The amount added was small enough (i.e. from 69 to 486 µl l^−1^) not to alter the diet’s overall nutritional content significantly. In control groups, an equivalent volume of distilled water was added to the diet to ensure that any effects observed were due to chlorothalonil exposure rather than variation in moisture content. Food was provided ad libitum to all flies throughout the experimental period.

*Drosophila* eggs were collected from the laboratory colony on grape juice agar plates (90 × 15 mm Petri dishes containing 15 g agar, 126 ml red grape juice, 6 g sucrose and 376 ml water), lightly coated with baker’s yeast paste, inside the population cage, allowing flies to oviposit for 18 h. Using a soft paintbrush, 50 eggs were collected and transferred in groups into Petri dishes (35 × 10 mm), each containing 3 ml of standard sugar–cornmeal yeast medium containing different concentrations of chlorothalonil (0, 50, 100, 150, 200, 250, 300 and 350 mg kg^−1^).

### 2.3. Effect of chronic exposure to chlorothalonil on larval survival

Larval survival was assessed by counting the number of pupae in each dish over 5 days, starting on the fourth day after egg deposition. Based on pupation rates, exposure to sublethal concentrations (less than or equal to 120 mg kg^−1^ chlorothalonil) was identified for subsequent experimental steps, focusing on the non-lethal effects of chronic chlorothalonil exposure.

### 2.4. Effect of chronic larval exposure to chlorothalonil on adult development and reproduction

#### 2.4.1. Adult body weight

To investigate the effects of chlorothalonil exposure on adult body weight, 50 eggs were collected from the laboratory colony and transferred into 10 ml of standard sugar–cornmeal yeast medium in Petri dishes (60 × 15 mm) with chlorothalonil concentrations of 0, 5, 10, 20, 40, 60, 80, 100 and 120 mg kg^−1^. Upon adult emergence, males and females were separated daily and transferred to vials containing the standard sugar–cornmeal yeast medium, with a total of 30 flies per vial. Flies were allowed to mate in these vials for 48 h, following Byrne and Rice’s method [77]. Ten-day-old mated males and females were subsequently collected, individually placed in 10 ml glass tubes, freeze-killed and dried in a drying oven at 60°C for 48 h. Each dried fly was then individually weighed on a Sartorius^®^ ME5 scale with 0.0001 µg precision. Five replicates per treatment, each consisting of two flies, were used for the body weight assessment.

#### 2.4.2. Number of ovarioles

To assess the impact of chlorothalonil on female reproductive output, 10-day-old females were sampled after larval development on substrates containing the different chlorothalonil concentrations, as described in the previous protocol. These females were euthanized at −20°C and preserved in 70% ethanol, with 25 females sampled per treatment. The choice of this age aligns with findings that *D. melanogaster* females reach peak egg production on day 10 of their life cycle [78,79]. Ovaries were dissected using Dumont forceps (Size 5, Inox 08) in a 1× phosphate-buffered saline (PBS) solution. The number of ovarioles present in one ovary was counted in 1× PBS under a dissecting microscope (John Morris, Motic SMZ-171) using a tungsten needle for precision.

#### 2.4.3. Female egg production

To assess the effect of chlorothalonil on fecundity, newly eclosed adults (10 females and 10 males) from the protocol outlined previously were sorted daily and transferred into vials containing the standard sugar–cornmeal yeast medium. Pools of five 3-day-old mated females were then placed into plastic insect cages with 3 ml of standard sugar–cornmeal yeast medium contained in 30 × 15 mm Petri dishes, with five replicates per treatment. The Petri dishes were replaced every 24 h, and the total number of eggs was counted under a dissecting microscope for 10 days. This period was selected as egg production within the first 10 days of adult life, which is widely regarded as a reliable indicator of lifetime egg production in *Drosophila* [80].

### 2.5. Ferrozine assay to detect the mitochondrial mitoferrin activity in male flies

In *Drosophila*, the mitochondrial iron transporter mitoferrin (*dmfrn*) is crucial for maintaining mitochondrial iron homeostasis, a process fundamental to spermatogenesis and sperm maturation. Iron levels in *Drosophila* can be quantified as a proxy for mitoferrin activity using a spectrophotometric method at 562 nm with a ferrozine reagent. This approach, known as the ferrozine assay, calibrates absorbance measurements against a standard curve, offering a reliable evaluation of iron metabolism and mitochondrial function in flies [72,81].

The ferrozine assay was conducted to quantify iron levels in male flies based on Missirlis *et al.* [82], with minor adjustments [83]. Twelve 10-day-old adult male flies per treatment were collected in fresh microcentrifuge tubes, euthanized at −20°C and homogenized in 250 μl of lysis buffer (Sigma-Aldrich (Australia) L1043-500RXN 10× Single Cell Lysis & Fragmentation Buffer) using a pestle. The homogenates were centrifuged twice at 16 000 r.p.m. for 5 min at 4°C, yielding an 80 μl sample. For each sample, 17 μl of concentrated HCl was added; a control was prepared by adding 17 μl of concentrated HCl to the lysis buffer alone. Both solutions were heated at 95°C for 20 min and centrifuged at 16 000 r.p.m. for 2 min. The resulting supernatant (66 μl) was transferred to a new tube and re-centrifuged at 16 000 r.p.m. for 2 min. From this, 50 μl of supernatant was combined with 20 μl of 75 mM ascorbate, mixed and spun down. Then, 20 μl of 10 mM ferrozine was added, vortexed and spun down. Finally, 40 μl of ammonium acetate buffer (pH 4.5, 2.5 M) was added, and the mixture was vortexed. The absorbance of each sample was measured at 562 nm using a 96-well microplate reader (SpectraMax Molecular Devices), and the mean absorbance per fly was calculated. Four replicates were performed for each treatment, with absorbance directly reflecting the iron concentration in each fly [72,81].

### 2.6. Feeding assay to quantify larval food intake

Third-instar larvae were reared on a standard fly diet and collected using a soft paintbrush and washed in PBS for 30 s. A total of 50 larvae, starved for 6 h, were transferred into 400 µl of feeding-assay media containing various concentrations of chlorothalonil (0, 5, 10, 20, 40, 60, 80, 100 and 120 mg kg^−1^ dissolved in distilled water), 20% yeast paste and 5% brilliant blue food dye (E133, colour index number 42090), with five replicates per treatment. Feeding assay media without dye and chlorothalonil served as a blank control. Fly tubes were sealed with laboratory film and incubated for 1 h [84]. Following exposure, larvae were washed in PBS, freeze-killed at −20°C and their digestive tracts were dissected in cold 1× PBS under a dissecting microscope (John Morris, Motic SMZ-171). For each replicate, five intact digestive tracts were transferred to 1.5 ml microcentrifuge tubes containing 0.75−1 mm glass beads and 50 µl of 1× PBS. These samples were homogenized using a Precellys^®^ 24 grinder for 5 min. Lysates were centrifuged at 15 000 r.p.m. for 5 min, and absorbance was measured at 630 nm using a Thermo Scientific™ NanoDrop™ 1000 UV-Vis spectrophotometer. The mean absorbance per larva was calculated, allowing for dye content quantification based on Keita *et al.* [84]. The amount of food intake was determined using a standard curve, prepared by serially diluting a blue dye sample in PBS at concentrations of 0.00, 0.02, 0.04, 0.06, 0.08, 0.10, 0.12, 0.14, 0.16, 0.18 and 0.20 µg ml^−1^, with five replicates per concentration.

### 2.7. Statistical analyses

The total number of pupae was analysed using a generalized linear model (Poisson distribution) with the concentration of chlorothalonil (linear and quadratic effect) as a continuous variable. The number of daily larvae was analysed using a generalized linear model (negative binomial distribution) with the concentration of chlorothalonil (linear and quadratic effect) and day as continuous variables. The lethal concentration that results in a response of 50% (LC50) was calculated using a dose–mortality curve (probit regression) at a 95% confidence level. The daily number of pupae was analysed using a subset of the initial dataset that did not contain the concentrations of chlorothalonil (greater than or equal to 250 mg kg^−1^), for which only a very low number of larvae were pupated.

Male and female body weights (log-transformed) were analysed using a linear model with the concentration of chlorothalonil (linear and quadratic effect for female weight and linear only for male weight) as a continuous variable. The number of ovarioles was analysed using a generalized linear model (Poisson distribution) with the concentration of chlorothalonil (linear effect) as a continuous variable. The total number of eggs produced after 10 days was analysed using a generalized linear model (Poisson distribution) with the concentration of chlorothalonil (linear and quadratic effect) as a continuous variable. Food intake was analysed using a linear model with the concentration of chlorothalonil (linear effect) as a continuous variable. The iron level was analysed using a linear model with the concentration of chlorothalonil (linear, quadratic and cubic effects) as a continuous variable. To compare and select the models that include the linear, quadratic and cubic effects of the chlorothalonil dose (nested model comparison), we used an ANOVA with the regression objects as separate arguments. The Akaike information criterion (AIC) of the models was also investigated. Residuals and Q–Q plots were examined for each model. Over- and under-dispersion were checked using the DHARMa package [85]. Trend lines were fitted using the loess (nonlinear) or lm (linear) arguments in R. All analyses were performed in R v. 4.3.1 (https://www.r-project.org) and RStudio 2023.06.2 build 561 (2009−2023 Posit Software, PBC). Graphs were created using the ‘ggplot2’ package [86]. For the survival analyses, graphs were created using MedCalc statistical software for the probit analysis.

## 3. Results

### 3.1. Larval survival

**Figure 1.**
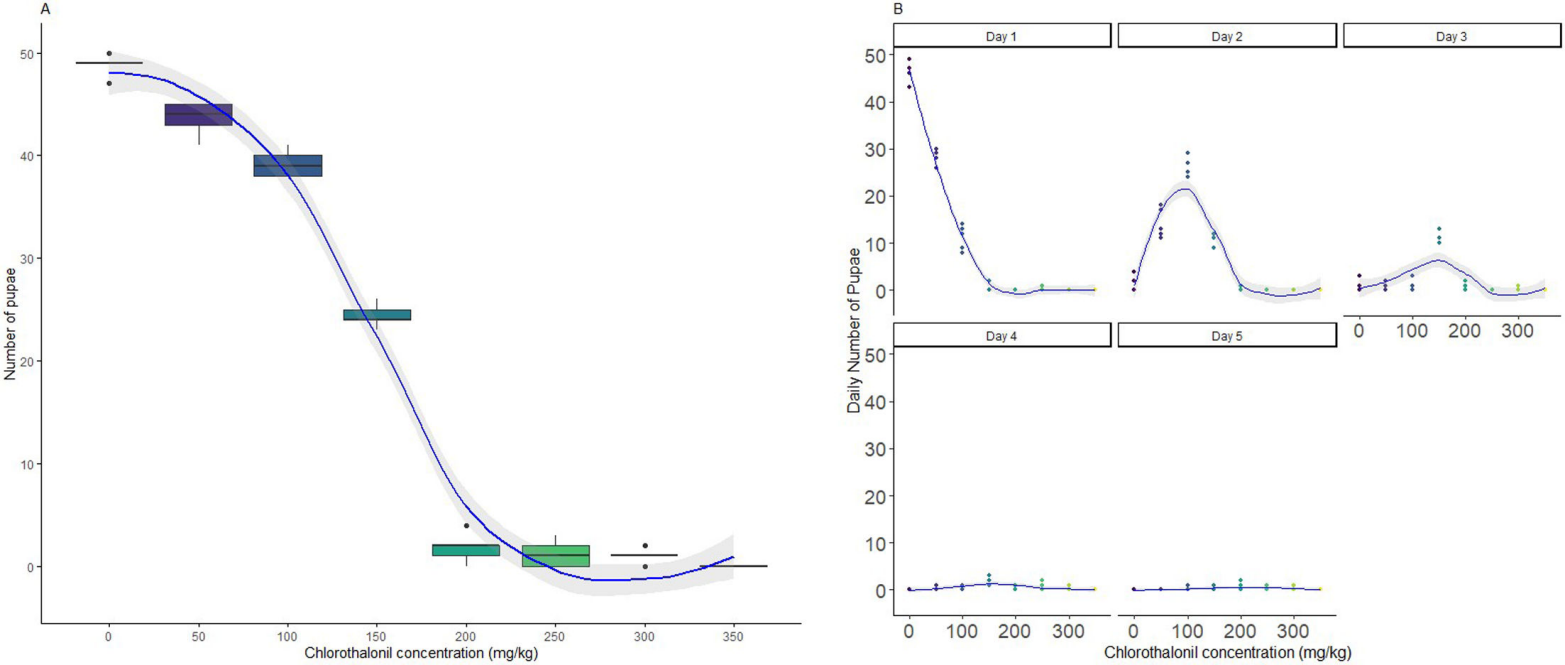
Effect of chronic larval exposure to chlorothalonil (0 to 350 mg kg^−1^ substrate) on the total and daily number of *Drosophila melanogaster* pupae. Cumulative number of pupae for Drosophila larvae fed seven concentrations of chlorothalonil (A) and daily number of pupae over 5 days for Drosophila larvae fed seven concentrations of chlorothalonil (B). Each dot represents an individual data point, and the shaded blue area indicates the 95% confidence interval around the local polynomial (loess) regression line.

The concentration of chlorothalonil in the substrate significantly affected the total number of pupae (*p* < 0.0001, electronic supplementary material, table S2). As chlorothalonil concentration increased, the number of pupae decreased (figure 1A). Further, at high concentrations (250 mg kg^−1^ and above), most larvae remained as larvae and failed to pupate (figure 1B). Additionally, there was an interaction between chlorothalonil concentration and day that influenced the daily larval count (*p* = 0.013, electronic supplementary material, table S2). Compared with the control group, larvae exposed to chlorothalonil took longer to pupate (figure 1B). While larvae in the control group mostly pupated on day 5, larvae exposed to 100 and 150 pupated on day 6 and 7, respectively. The chronic LC50 for chlorothalonil was determined as 13.714 mg kg^−1^ of substrate (electronic supplementary material, figure S1).

### 3.2. Adult body weight

**Figure 2.**
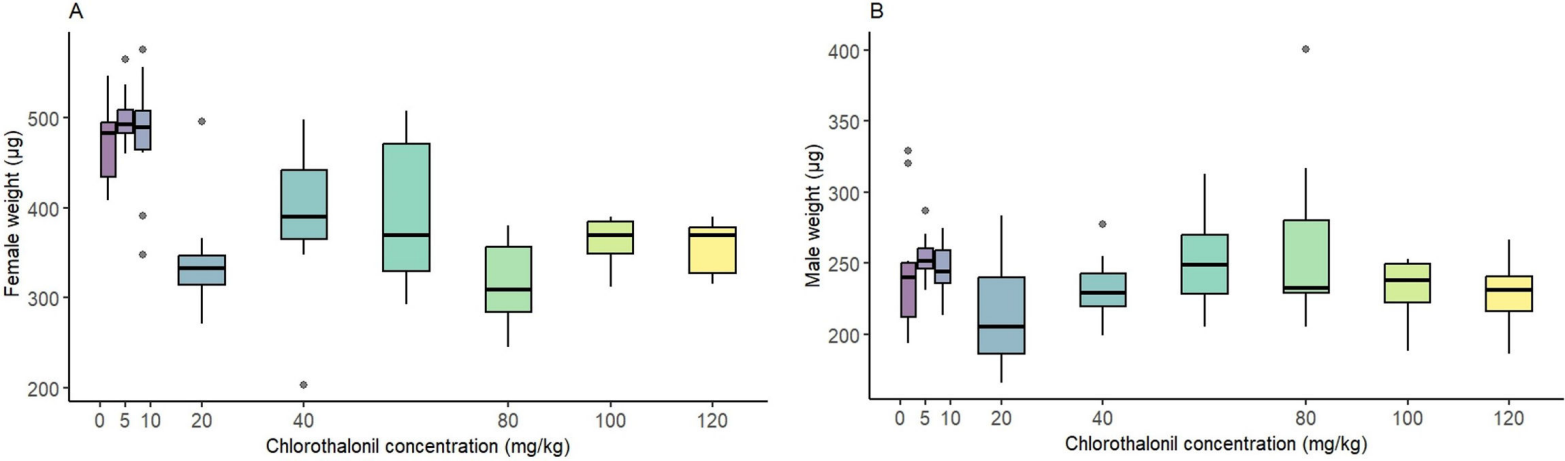
Effect of sublethal, chronic exposure to chlorothalonil (0 to 120 mg kg^−1^ substrate) on the body weight of adult *Drosophila melanogaster*. Effect of chlorothalonil exposure on female weight (A) and male weight (B). Boxplots represent the distribution of weight across different concentrations of chlorothalonil. Dots represent outliers.

Chlorothalonil exposure significantly affected the body weight of female flies (*p* < 0.001, electronic supplementary material, table S2), with a noticeable decrease observed after exposure to 10 mg kg^−1^ of chlorothalonil per substrate (figure 2A). In contrast, chlorothalonil exposure did not influence the body weight of male flies (*p* = 0.55, figure 2B, electronic supplementary material, table S2).

### 3.3. Number of ovarioles

**Figure 3.**
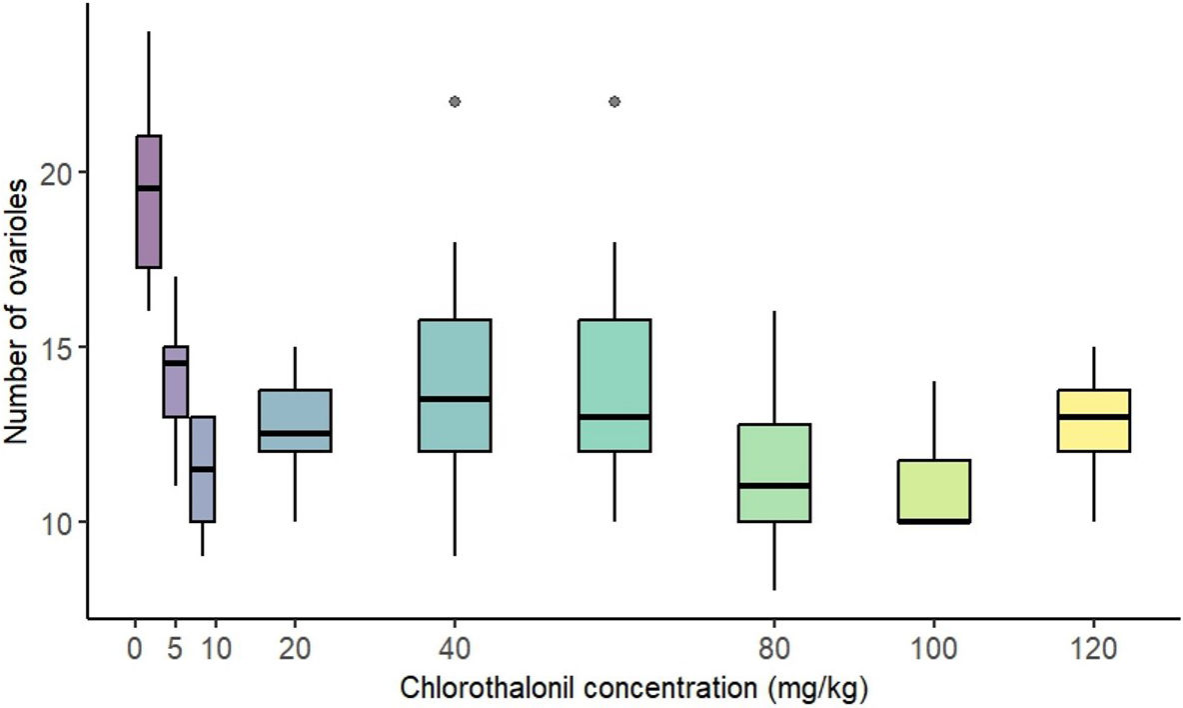
Effect of sublethal, chronic exposure to chlorothalonil (0 to 120 mg kg^−1^ substrate) on the number of ovarioles of *Drosophila melanogaster* females. Effect of chronic exposure to chlorothalonil on the number of ovarioles in 10-day-old females. Boxplots represent the distribution of ovariole counts across different concentrations of chlorothalonil. Dots represent outliers.

Exposure to chlorothalonil during the larval stage significantly affected the number of ovarioles in adult females (*p* = 0.001, electronic supplementary material, table S2). The model estimate indicates a decrease of −log 0.0023 in ovariole count with each unit increase in chlorothalonil concentration (figure 3).

### 3.4. Female egg production

**Figure 4.**
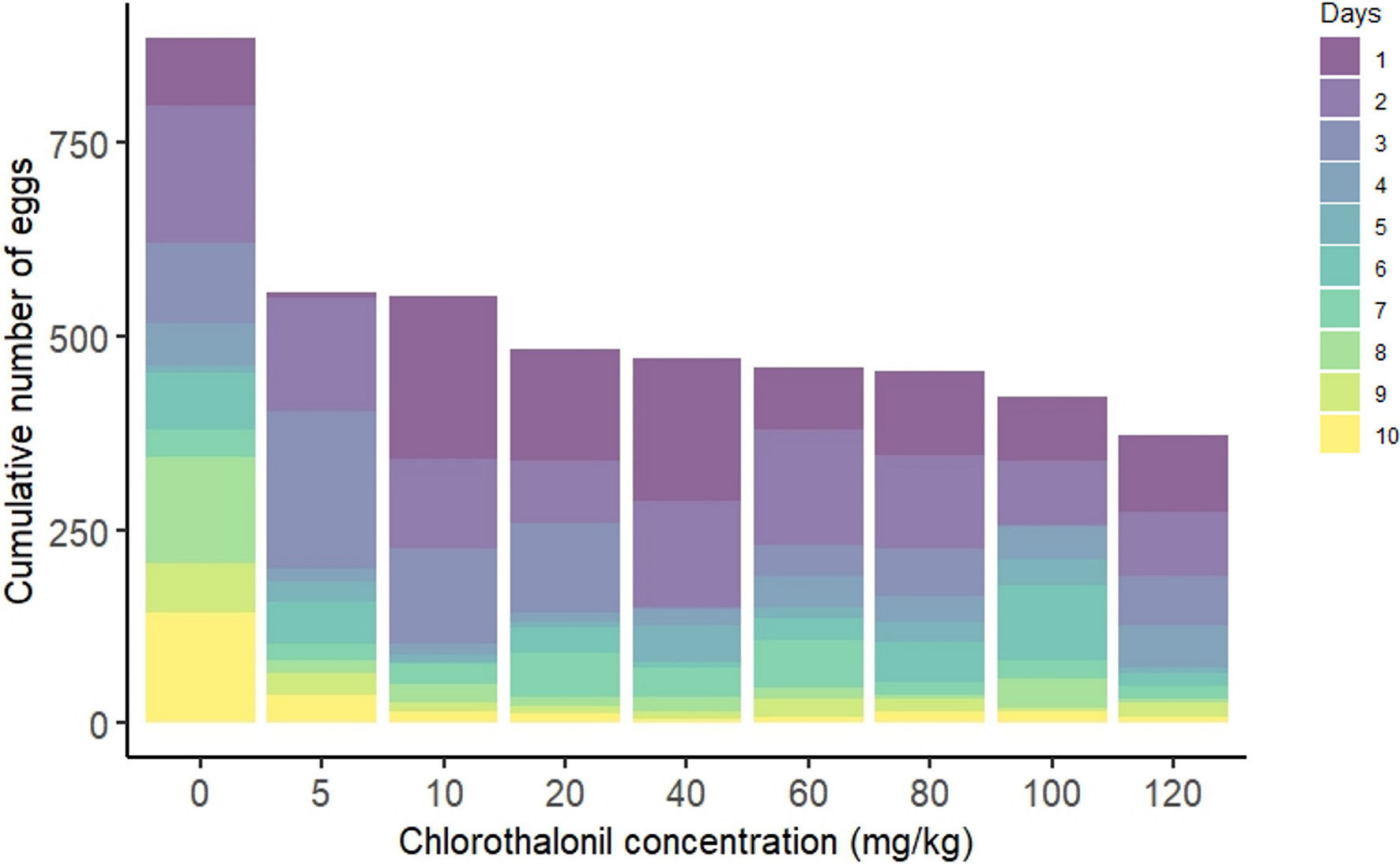
Effect of sublethal, chronic larval exposure to chlorothalonil (0 to 120 mg kg^−1^ substrate) on the cumulative number of eggs produced by *Drosophila melanogaster* over 10 days. Effect of chronic exposure to chlorothalonil on the average number of eggs produced by pools of five females over a 10-day period. Each bar is segmented by shades representing each day (1 to 10), showing the daily contribution to the cumulative number of eggs for each chlorothalonil concentration level.

The number of eggs laid by female flies over 10 days significantly declined with increasing chlorothalonil concentrations (*p* < 0.0001, figure 4, electronic supplementary material, table S2). Females exposed to the highest chlorothalonil concentration during the larval stage (120 mg kg^−1^ of substrate) laid 58% fewer eggs compared with the control group (figure 4). Even at the lowest chlorothalonil concentration tested (5 mg kg^−1^ of substrate), there was a 37% reduction in egg production compared with the control group.

### 3.5. Ferrozine assay

**Figure 5.**
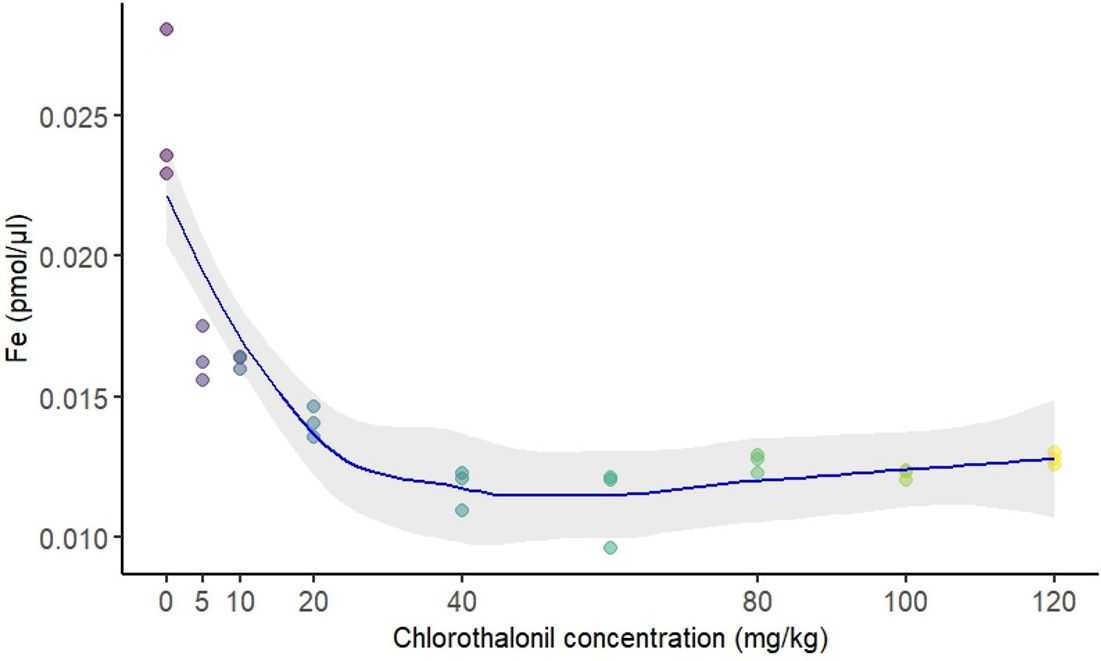
Effect of sublethal, chronic larval exposure to chlorothalonil (0 to 120 mg kg^-1^ substrate) on iron content in male *Drosophila melanogaster*. The figure displays the effect of chronic chlorothalonil exposure during the larval stage on iron content (Fe) in 10-day-old male flies. Dots represent single data points. The grey shaded area represents the 95% confidence interval.

Iron levels in male flies were significantly affected by larval exposure to chlorothalonil (*p* = 0.0003, electronic supplementary material, table S2). Iron levels in adult males dropped sharply following larval exposure to 5 mg kg^−1^ of chlorothalonil (figure 5). At higher exposure levels (40–120 mg kg^−1^ of substrate), iron levels stabilized at roughly half the amount observed in control flies (figure 5).

### 3.6. Food intake

**Figure 6.**
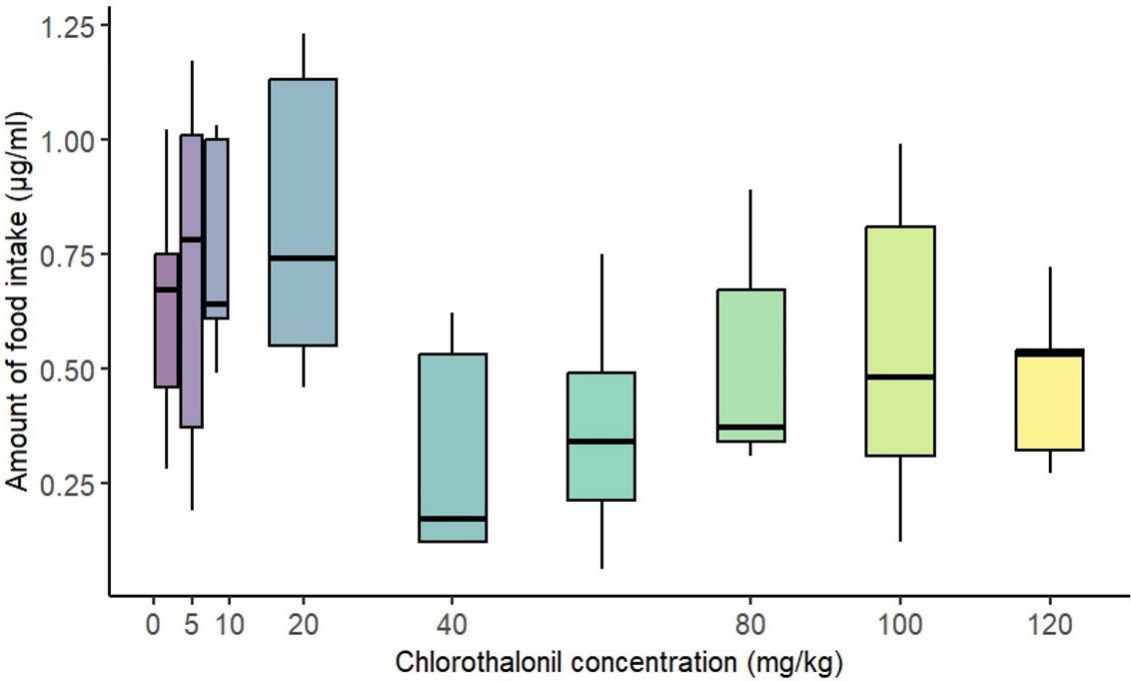
Effect of sublethal, chronic larval exposure to chlorothalonil (0 to 120 mg kg^-1^ substrate) on food intake in *Drosophila melanogaster* third instar larvae. The figure displays the effect of chlorothalonil exposure on food intake for one hour by fly larvae.

Chlorothalonil exposure did not significantly influence larval food intake (*p* = 0.056, electronic supplementary material, table S2). However, there was a slight trend indicating that larvae exposed to 40 mg kg^−1^ or higher concentrations of chlorothalonil consumed slightly less food compared with those exposed to lower concentrations (figure 6).

## 4. Discussion

In this study, we showed that chronic exposure to chlorothalonil affects several life history traits of *D. melanogaster*. We observed a dose-dependent increase in larval mortality, with concentrations of 200 mg kg^−1^ or higher causing complete mortality and exposure to a dose of 60.98 mg kg^−1^ resulting in a 50% mortality rate (LC50). These findings align with previous research on honeybees [87–89]. Chlorothalonil exposure also prolonged developmental time and decreased fecundity, suggesting that exposure may disrupt critical processes during larval development and adult life. To better understand the effects of chlorothalonil on pupal mortality as observed in this study, it would be interesting to investigate how chlorothalonil affects egg hatching rate and larval development between instars.

The negative impact of chlorothalonil on *Drosophila* reproductive output was evident in the reduced number of ovarioles in females, a trait strongly correlated with egg production [90,91]. Ovariole formation occurs during larval development through the morphogenesis of terminal filaments (TFs), which are essential somatic structures that establish the future number of ovarioles at the larval–pupal transition [92]. Chemical exposure, such as chlorothalonil, could disrupt TF development, reducing ovariole numbers and subsequently limiting reproductive capacity. Decreases in ovary size, although not systematically measured here, were also observed, suggesting further exploration of chlorothalonil’s impact on hormone production, particularly follicle-stimulating hormone and oestrogen receptor activity, which are altered in mammalian models [93,94].

A substantial reduction in egg production was observed in females exposed to chlorothalonil as larvae, with over a 50% decrease in the number of laid eggs at 120 mg kg^−1^ and clear reductions at concentrations as low as 5 mg kg^−1^. Reduction in egg laying could result from sperm depletion; however, *D. melanogaster* females are known to store and utilize sperm for at least two weeks following mating [95–97]. Typically, between 700 and 1000 sperm are retained in the seminal receptacle and spermathecae, enabling continuous fertilization without the need for remating [96,98,99]. Although sperm numbers gradually decline over time, this stored quantity was certainly sufficient to sustain reproductive output over the 10 day fecundity assay used in this study. Therefore, the observed reduction in egg production is more likely to be due to chlorothalonil-induced physiological disruptions rather than limited sperm.

Egg production in *D. melanogaster* is regulated by a complex interplay of internal and external factors, with hormonal regulation including juvenile hormone, insulin-like growth factors, ecdysone and nutrition being two critical determinants of reproductive success. Given chlorothalonil’s known oral toxicity, we hypothesized that dietary exposure would lead to a reduction in the feeding rate or act as a feeding deterrent, thereby affecting larval development. However, we could only detect a slight decrease, but not a significant decrease in food intake, even at high chlorothalonil concentrations. This suggests that reduced nutrient intake alone does not fully explain the observed decline in egg production. It is important to note that the feeding assay measured food consumption over 1 h, and extending the assay duration may reveal delayed or cumulative effects on larval feeding behaviour. It remains uncertain whether chlorothalonil exposure alters larval metabolism, potentially leading to long-term physiological consequences. Additionally, chlorothalonil may disrupt the endocrine balance of exposed flies, redirecting energy resources from reproduction to survival [100]. In response to stressors such as chlorothalonil, females may suppress egg production due to elevated ecdysone levels, which in turn reduce ecdysone-triggering hormone levels, thereby inhibiting juvenile hormone activity [101]. Elevated steroid hormone levels can result in oogenesis arrest and impaired neuronal signalling, particularly within the octopaminergic system, which plays a crucial role in regulating ovulation and reproductive tract function [102]. Furthermore, oxidative stress and metabolic disruptions induced by chlorothalonil exposure may further exacerbate these hormonal imbalances, leading to additional reproductive suppression. Chlorothalonil exposure may therefore disrupt endocrine regulation, resulting in a shift in energy allocation from reproduction to survival and impairing neurohormonal signalling, ultimately leading to a significant reduction in reproductive output.

Female body weight was also significantly reduced following chlorothalonil exposure, especially at concentrations above 20 mg kg^−1^, which may correlate with the observed reduction in the number of ovarioles. A weak but statistically significant positive correlation was observed (Pearson’s *r* = 0.271, *p* = 0.01), suggesting that higher ovariole numbers are modestly associated with higher body weight (electronic supplementary material, figure S2). Body size and reproductive output are known to be positively correlated in *Drosophila* and other insects [103,104], indicating that chlorothalonil exposure during larval development may impair overall fitness in adult females. Female insects may exhibit greater sensitivity to toxicants due to their higher energetic investment in reproduction [99], larger body size and lipid content, and potential differences in detoxification enzyme activity [105]. The observed sex-specific effect, where only females exhibited weight loss, suggests a physiological difference in response to chlorothalonil that requires further investigation.

Male reproductive health was assessed through body iron content, which was found to decrease in response to chlorothalonil exposure. Iron is a vital micronutrient involved in enzyme activity and cellular respiration. In *Drosophila*, iron homeostasis is regulated by mitoferrin, a mitochondrial iron transporter that is crucial for spermatogenesis and sperm maturation [106,107]. Impaired mitoferrin activity can lead to structural changes in the testes, such as reduced testis length [72,108]. The reduction in iron content observed in this study suggests that chlorothalonil may affect male fertility by interfering with mitoferrin activity and iron metabolism. Further studies are needed to explore the effects of chlorothalonil on sperm morphology and function to fully understand its impact on male reproductive health.

Chlorothalonil exerts its toxicity by targeting sulfhydryl (thiol, –SH) groups in proteins and glutathione (GSH), a critical non-protein thiol in aerobic cells. Sulfhydryls are essential components of cellular redox systems, mitigating oxidative damage by reducing reactive oxygen species (ROS) generated during metabolic processes such as cellular respiration [109–111]. GSH plays a key role in maintaining the redox balance, detoxifying xenobiotics and ensuring cellular homeostasis. However, chlorothalonil disrupts this sulfhydryl equilibrium, resulting in oxidative stress, tissue damage and cellular dysfunction caused by excessive ROS accumulation [109–113].

In insects, chlorothalonil toxicity can occur directly through binding to GSH or indirectly by inhibiting glutathione S-transferase (GST), an enzyme critical for xenobiotic detoxification. Interestingly, detoxification capacity may vary across species, as honeybees possess only one GST-encoding gene compared with *Drosophila*’s 38, potentially making honeybees more susceptible to chlorothalonil [114]. Despite *Drosophila*’s extensive GST gene family, larvae demonstrated high sensitivity to chlorothalonil exposure.

Although this study did not investigate the effects of chlorothalonil on oxidative stress, antioxidative response, or glycolytic enzyme impairment in *D. melanogaster*, previous research suggests that chlorothalonil exposure can induce oxidative damage, disrupt antioxidant defence mechanisms and impair glycolytic enzyme activity in other organisms such as amphibians and mammals [44,93]. Beyond disrupting sulfhydryl equilibrium, chlorothalonil affects cellular respiration in fungi by targeting mitochondrial complexes, particularly the electron transport chain, impairing energy production and metabolism [115]. Additionally, chlorothalonil inhibits glyceraldehyde-3-phosphate dehydrogenase (GAPDH), a highly conserved glycolytic enzyme essential for NADH production [92,116–118]. As GAPDH is a highly conserved housekeeping protein present across all domains of life, its inhibition by chlorothalonil highlights the chemical’s interference with fundamental metabolic pathways in a wide range of organisms [116]. These combined effects highlight chlorothalonil’s profound impact on essential physiological processes in non-target species.

Chronic larval exposure to chlorothalonil has significant adverse effects on *D. melanogaster* at the larval and adult stages, affecting survival, development and fecundity. *Drosophila* is not only a model system widely used in laboratory experiments, but species of *Drosophila* are also widely present in orchards and other agricultural systems where they forage and lay eggs [119–121]. While the direct toxicological effects of chlorothalonil on *Drosophila* are well-established, its indirect impact through the disruption of yeast and other microbial communities in the environment, which are critical nutritional and symbiotic sources, may also contribute to developmental and reproductive impairments. This often-overlooked impact could have broader implications for non-target insect populations exposed to fungicide-contaminated environments. These findings emphasize the potential risks chlorothalonil poses to Drosophila and, more widely, insect populations, even at low doses, and underscore the importance of assessing non-target effects in broader ecological contexts, particularly for chemicals widely used in agriculture.

Amid growing concerns over pesticide-induced insect decline and environmental pollution, interest in sustainable biopesticides has increased, with a particular focus on botanical fungicides derived from plant-based compounds. These natural metabolites offer effective disease control while exhibiting low toxicity to non-target organisms, making them strong candidates for environmentally friendly pest management [122]. Additionally, their compatibility with integrated pest management programmes promotes biodiversity conservation by preserving beneficial insect populations, including pollinators and natural enemies. Collectively, these attributes highlight the value of biopesticides in advancing ecologically responsible and sustainable agricultural practices.

## Data Availability

Data and R codes, as well as raw data, have been uploaded as electronic supplementary material [123].
